# Survival of *Salmonella* Typhimurium in Vacuum-Sealed Maize Tortillas During Refrigerated and Ambient Storage

**DOI:** 10.3390/foods15173103

**Published:** 2026-09-01

**Authors:** Jesús Andrés Torres-Vélez, Anai Zavala-Franco, Beatriz L. Álvarez-Mayorga, Marcela Gaytán-Martínez, Eduardo Morales-Sanchez

**Affiliations:** 1 CICATA-IPN Unidad Querétaro, Instituto Politécnico Nacional, Querétaro C.P. 76090, Mexico; vandrest27@gmail.com; 2 Facultad de Química, Universidad Autónoma de Querétaro, Santiago de Querétaro C.P. 76010, QRO, Mexico; anai.zavala.f@gmail.com (A.Z.-F.); beatriz.alvarez@uaq.edu.mx (B.L.Á.-M.); marcela.gaytan@uaq.mx (M.G.-M.)

**Keywords:** maize tortillas, vacuum-sealed, *Salmonella* survival, coliforms, Weibull model

## Abstract

As dietary habits evolve, cereal-based foods, such as maize tortillas, are increasingly consumed as ready-to-eat products, yet their physicochemical properties may influence microbial stability during storage. This study characterized the microbiological and physicochemical properties of commercial vacuum-sealed maize tortillas and evaluated *Salmonella enterica* survival in vacuum-sealed laboratory-prepared maize tortillas. Nine commercial brands were characterized by proximate composition, moisture content, water activity (0.945–0.965), and pH (4.76–5.50), confirming a high-moisture and carbohydrate-rich matrix. Indicator microorganisms revealed moderate contamination levels, with total coliforms exceeding regulatory limits in several samples. Challenge studies demonstrated that *S.* Typhimurium survival at 4 and 25 °C followed non-linear inactivation kinetics accurately described by the Weibull model (R^2^ > 0.90). Storage temperature significantly affected the shape parameter (*p* = 0.026), whereas packaging conditions significantly influenced the estimated 5-log reduction time (t_5_). Vacuum-sealed tortillas exhibited prolonged pathogen survival, particularly under refrigeration (>60 days), where bacterial populations remained detectable for extended storage periods. These findings challenge the assumption that vacuum-sealing improves microbiological safety in cereal-based foods.

## 1. Introduction

Cereal-based foods constitute a major component of the human diet worldwide and are increasingly commercialized as extended-shelf-life ready-to-eat (RTE) products. To improve product stability and safety, the food industry has implemented preservation technologies such as thermal processing, modified atmosphere packaging, and the use of preservatives [1]. Recent studies have demonstrated the effectiveness of these approaches in improving the quality, safety, and shelf life of food products, including vacuum-sealed chicken legs and refrigerated chicken patties [2,3]. Among these, vacuum-sealing has gained increasing attention as a cost-effective preservation technology that reduces oxygen availability, thereby limiting oxidative reactions and delaying the growth of aerobic spoilage microorganisms [4]. However, oxygen reduction under these conditions does not necessarily inhibit facultative anaerobic or stress-adapted microorganisms and therefore cannot be considered sufficient to guarantee microbiological safety [5,6].

*Salmonella* remains one of the leading causes of bacterial gastroenteritis worldwide. It exhibits a remarkable ability to survive under adverse environmental conditions, particularly in low-moisture and carbohydrate-rich foods, including cereals and cereal-derived products. In such matrices, reduced metabolic activity and enhanced stress-tolerance mechanisms may favor long-term survival rather than active growth [7]. This persistence is a significant food safety concern, particularly for RTE products that do not undergo additional lethal processing before consumption [8]. Despite the growing commercialization of vacuum-sealed cereal products, quantitative information regarding *Salmonella* survival kinetics in vacuum-sealed maize tortillas remains limited.

Industrialized maize tortillas are a staple food in Mexico and are increasingly commercialized in national and international markets. These products constitute a complex carbohydrate-rich matrix characterized by high moisture content, intermediate water activity, and a starch–protein network formed during thermal processing. Industrial formulations frequently incorporate hydrocolloids and dietary fiber, which may modify water distribution and generate microenvironments that enhance microbial persistence [9,10]. Previously, some studies have demonstrated that oxygen reduction extended the shelf life of wheat flour tortillas, particularly under refrigeration [11]. However, information regarding the behavior of *Salmonella* in vacuum-sealed maize tortillas remains scarce.

Microbiological inactivation in heterogeneous food matrices rarely follows log-linear behavior due to physiological heterogeneity and stress adaptation phenomena within microbial populations, which can generate resistant subpopulations; therefore, flexible non-linear models such as the Weibull have been widely applied to characterize microbial inactivation by accounting for physiological heterogeneity and the presence of resistant subpopulations, thereby providing a more realistic description of survival dynamics than conventional first-order models [12,13]. Such predictive approaches are particularly valuable for microbial risk assessment and for designing evidence-based preservation strategies for cereal-based foods.

Therefore, this study aimed to characterize the physicochemical and microbiological quality of commercially available vacuum-sealed maize tortillas and to evaluate *Salmonella enterica* survival kinetics in laboratory-prepared maize tortillas as a controlled food matrix model under vacuum and non-vacuum conditions during refrigerated and ambient storage using the Weibull model.

## 2. Materials and Methods

### 2.1. Sample Collection

Vacuum-sealed maize tortillas available in supermarkets in the city of Querétaro were surveyed between January and February 2024. Six independent packages from each of nine commercial brands of maize tortillas were selected, with declared shelf lives of longer than one month, as verified by the production and expiration dates on the batch code. Samples were transported to the laboratory and stored at 4 °C until physicochemical and microbiological analyses were performed.

### 2.2. Chemical Composition of Vacuum-Sealed Commercial Tortillas

The pH of tortillas was measured according to AOAC method 02-52 (2002) using a calibrated pH meter (HI2211, Hanna Instruments, Woonsocket, RI, USA). Water activity (a_w_) was determined using a water activity meter (AquaLab, Pullman, WA, USA) according to the AOAC method 978.18 (2002).

Proximate composition was determined for each brand according to AOAC (2002) official methods: moisture content (method 925.10), lipid content (method 920.39), protein content (method 2001.11), ash content (method 923.03), and dietary fiber (soluble and insoluble fractions) (method 991.43). Total carbohydrate content was calculated by difference. All proximate analyses were performed in triplicate, and results are expressed on a dry-weight basis.

### 2.3. Indicator Microorganisms

In a biosafety cabinet, 10 g of each sample was aseptically transferred into sterile polyethylene bags containing 90 mL of 0.1% peptone diluent and homogenized for 1 min. Serial decimal dilutions were then prepared, and 1 mL aliquots of the appropriate dilutions were surface-plated onto MC-Media Pad^®^ (Merck, Darmstadt, Germany; detection limit: 1 log CFU/g). The following indicators were analyzed: aerobic plate count (incubated at 35 ± 1 °C for 48 h), yeast and mold enumeration (incubated at 25 ± 1 °C for 72 h), and total coliforms and *Escherichia coli* (incubated at 35 ± 1 °C for 24 h), according to the manufacturer’s protocol.

### 2.4. Survival and Modeling Behavior of Salmonella in Vacuum-Sealed Maize Tortillas

#### 2.4.1. Culture Conditions, Inoculation, and Storage of Maize Tortillas

*Salmonella enterica* serovar Typhimurium (ATCC 14028) resistant to 200 ppm of rifampicin was used in the microorganism challenge test. A single strain, *Salmonella* Typhimurium ATCC 14028, was selected to maintain a standardized and controlled experimental system and to minimize variability associated with strain-to-strain differences in stress resistance and survival behavior. Rifampicin resistance served as a phenotypic marker to facilitate the selective recovery and enumeration of the inoculated strain in the presence of background microbiota [14]. The strain was activated in tryptic soy broth (TSB; Bioxon, SD, Benham Pharmaceuticals Ltd., Dhaka, Bangladesh) and incubated at 35 °C for 24 h. A second subculture was performed by inoculating fresh TSB with the initial culture and then incubating it at 35 °C for 18 h.

*S.* Typhimurium cells were harvested by centrifugation, washed twice with sterile isotonic saline solution (0.85% NaCl), and resuspended in the same solution. The inoculum concentration was determined by the spread plate method on tryptic soy agar (TSA; Bioxon, SD) supplemented with rifampicin (200 ppm), followed by incubation at 35 °C for 24 h.

For *S.* Typhimurium inoculation, four independent production batches of maize tortillas were produced in a laboratory, and, in a biosafety cabinet, 400 µL of the bacterial suspension (approximately 7 log CFU/mL) was uniformly distributed between two tortillas (20 g each). The inoculated tortillas were sandwiched between two additional tortillas to simulate commercial stacking conditions and dried for 30 min to restore the initial conditions (pH = 7.98 and a_w_ = 0.97), yielding an initial contamination level of approximately 6.5 log CFU/g. The inoculum level of 6.5 log CFU/g was selected to avoid the detection limit, ensuring that sufficient viable cells could be recovered throughout storage and to allow the survival kinetics and inactivation pattern to be adequately characterized over the experimental period.

To evaluate the combined effect of packaging conditions and storage temperature on pathogen survival, two batches were vacuum-sealed into commercial polyethylene bags (20 × 20 cm), which were thermally sealed using a double sealing line to prevent gas exchange, employing a MIGSA D70-600B vacuum sealer (Elite Migsa, Guadalajara, México). At the same time, the remaining two were packaged without vacuum conditions (Control). Subsequently, one vacuum-sealed batch and one non-vacuum batch were stored at 4 °C, whereas the other two batches (one vacuum-sealed and one non-vacuum) were stored at 25 °C.

#### 2.4.2. Quantification of *S.* Typhimurium Survival in Maize Tortillas

*S.* Typhimurium survival was quantified using three packages per storage condition. Traditional packaging was evaluated on days 0, 1, 3, 5, 7, 14, 21, 28, 35, and 49, while vacuum-sealed tortillas were evaluated on days 0, 1, 3, 5, 7, 14, 28, 49, 56, 63, and 70 at 4 °C. Under aseptic conditions, tortillas were torn into small pieces, and 10 g samples were transferred into sterile plastic bags. Subsequently, 90 mL of 0.1% (*w*/*w*) peptone diluent was added, and samples were homogenized. Decimal dilutions were prepared, and 100 µL aliquots were spread onto TSA supplemented with rifampicin (200 ppm) for selective recovery of the rifampicin-resistant *S.* Typhimurium strain. Plates were incubated at 35 °C for 24 h. Results are expressed as log CFU/g, considering the total weight of the stacked tortillas.

#### 2.4.3. Modeling *S.* Typhimurium Survival Kinetics by a Primary Model

To describe the inactivation kinetics of *S.* Typhimurium inoculated in maize tortillas packaged under traditional and vacuum conditions, survival data (log CFU/g) were fitted using the Weibull model (Equation (1)). The model includes three parameters: *N*_0_ (initial microbial population), δ (the time required for the first decimal reduction), and *p* (the shape parameter of the survival curve). Values of *p* < 1 indicate concave survival curves, whereas *p* > 1 indicates convex curves.

(1) $${log}_{10}N={log}_{10}N_{0}-{\left(\frac{t}{\delta }\right)}^{p}$$

Model performance was evaluated using the root-mean-square error (RMSE) and the coefficient of determination (R^2^). RMSE < 0.5 and R^2^ > 0.8 values indicate a better goodness-of-fit between predicted and observed microbial counts [14]. Finally, with the Weibull parameters, t_5_ (time to 5 decimal reductions) was estimated in each treatment, as previously described for food systems [15,16].

### 2.5. Statistical Analyses

Counts of indicator microorganisms (CFU/g) were transformed to decimal logarithms before statistical analysis. The distribution of the data was assessed using the Shapiro–Wilk test. As normality assumptions were not met, results are expressed as median (range), and differences among brands were evaluated using the Kruskal–Wallis test followed by Dunn’s post hoc test. Physicochemical parameters and Weibull model parameters are expressed as mean ± standard deviation. Physicochemical data were analyzed by one-way analysis of variance (ANOVA), followed by the Tukey–Kramer post hoc test to evaluate differences between brands.

For the analysis of Weibull model parameters (*N*_0_, δ, *p*, and t_5_), data were analyzed by two-way ANOVA (packaging type and storage temperature), and effect sizes were estimated to quantify the magnitude of the evaluated factors. Eta squared (η^2^) and omega squared (ω^2^) were calculated for each source of variation. The η^2^ statistic estimates the proportion of total variance explained by each factor, whereas ω^2^ provides a less biased estimate at the population level by accounting for sampling error. These metrics allow the interpretation of the practical relevance of each factor beyond statistical significance. Statistical significance was set at α < 0.05. All statistical analyses were performed using RStudio software (version 2024.04.1+748).

## 3. Results

### 3.1. Physicochemical Properties of Vacuum-Sealed Maize Tortillas

The physicochemical and proximate characteristics of the commercial maize tortillas are shown in Table 1. Moisture content varied considerably among brands (35.6–49.1%), whereas a_w_ remained consistently high (0.945–0.965), indicating that all products provided favorable conditions for microbial survival. pH ranged between 4.76 and 5.51, with V8 exhibiting the highest and V2 the lowest values. Regarding nutritional composition, protein content varied from 7.70 (V8) to 9.32 g/100 g (V5), while fat content ranged from 0.98 to 3.2 g/100 g in V6 and V2 brands, respectively. Dietary fiber showed the greatest variability among brands, ranging from 3.74 (V4) to 20.17 g/100 g (V3), reflecting differences in formulation, while carbohydrate content remained comparatively constant. Ash content varied between 1.65 and 3.03 g/100 g. Significant differences among brands (*p* < 0.05) were observed for all proximate parameters evaluated.

### 3.2. Microbiological Profile of Cold Maize Tortillas

The microbiological quality of the commercial vacuum-sealed maize tortillas is presented in Table 2. Aerobic plate count (APC) median values ranged from 2.03 to 2.88 Log CFU/g with no significant differences among brands. Molds and yeasts (M&Y) were generally detected at very low levels. Median counts remained below the detection limit (<1.00 Log CFU/g) in seven of the nine brands (V1–V7). Detectable counts were observed only in V8 (1.15 Log CFU/g) and V9 (1.30 Log CFU/g); however, these values were not significantly different from those of the other brands (*p* > 0.05). Finally, total coliform (TC) counts varied from 1.20 to 2.44 Log CFU/g. The highest median count was recorded for V6 (2.44 Log CFU/g), whereas V8 showed the lowest value (1.20 Log CFU/g). Statistical analysis showed significant differences among brands (*p* < 0.05).

Using the same enumeration plates, *Escherichia coli* was differentiated from total coliforms and detected in 3 of the 9 brands analyzed: V1 (median 1.00 log CFU/g; range <1–1.48), V5 (median 1.20 log CFU/g; range <1–1.60), and V9 (median 1.15 log CFU/g; range <1–1.48). The presence of this indicator microorganism suggests deficiencies in handling and packaging practices, as *E. coli* in foods is widely recognized as an indicator of fecal contamination. Because the presence of *E. coli* indicates a potential fecal contamination event and, consequently, the possible introduction of enteric pathogens such as *Salmonella*, its detection provides a microbiological basis for assessing the survival of *Salmonella* under relevant storage conditions. Because *Salmonella* was not detected in any of the commercial samples, it was proposed for evaluating pathogen survival following simulated post-process contamination in laboratory-prepared maize tortillas under vacuum-sealed and traditional packaging conditions representative of commercial storage.

### 3.3. Survival of S. Typhimurium During Storage of Vacuum-Sealed Maize Tortillas

**Figure 1:**
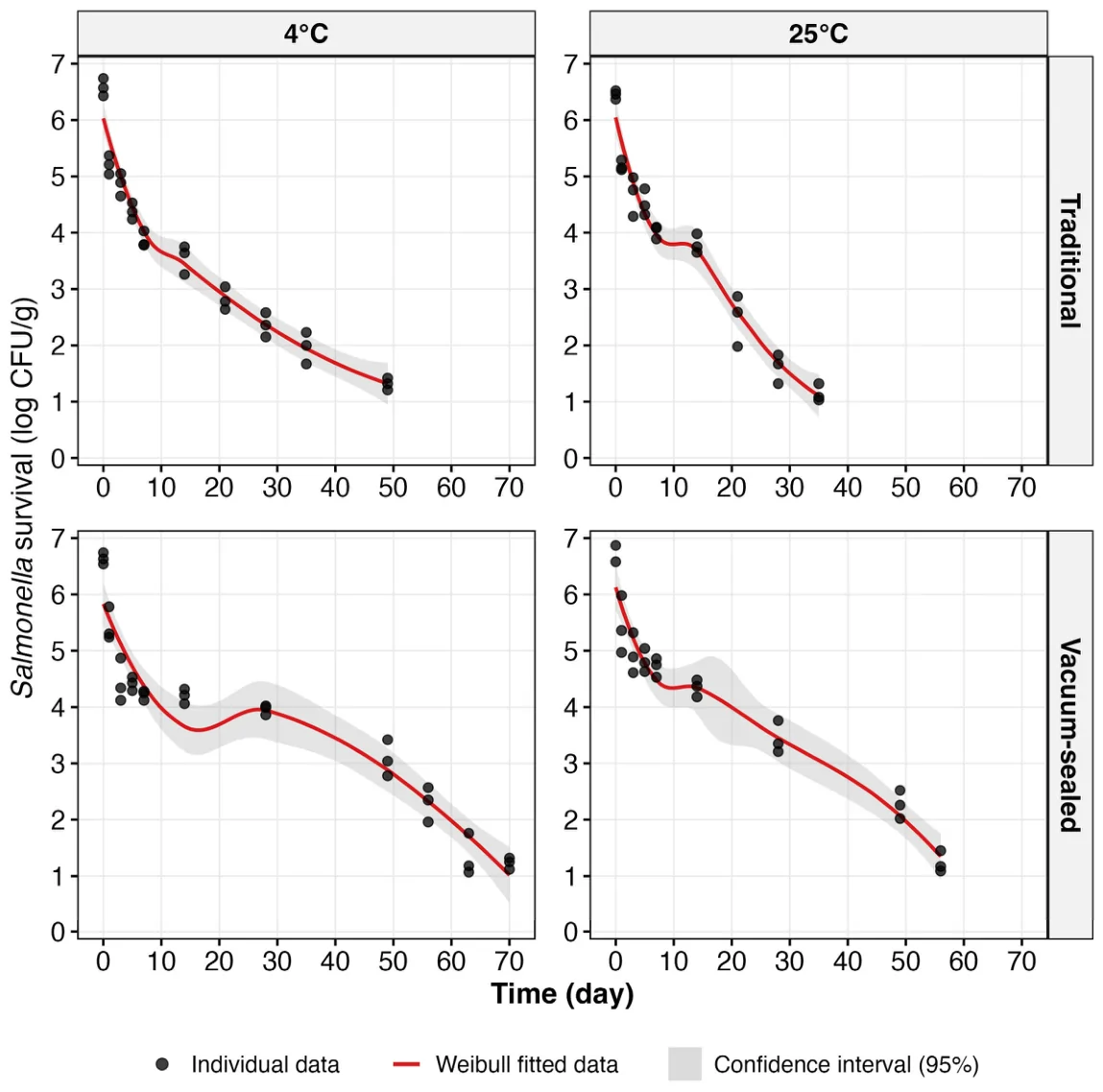
Survival curves of *S.* Typhimurium in maize tortillas under traditional and vacuum-sealing conditions during storage at 4 °C and 25 °C fitted with the Weibull model. Points represent experimental data (log CFU/g), red lines represent Weibull model fits, and shaded areas indicate 95% confidence intervals.

After characterizing the microbiological quality of the commercial samples, *S.* Typhimurium survival was evaluated in experimentally inoculated, laboratory-prepared maize tortillas packaged using either traditional or vacuum-sealed packaging and stored at 4 and 25 °C. The survival curves obtained under each storage condition are presented in Figure 1. In traditionally packaged tortillas, populations decreased from approximately 6.5 Log CFU/g to 1.0–1.5 Log CFU/g after approximately 45 days at 4 °C and 40 days at 25 °C. A similar decline was observed in vacuum-sealed tortillas; however, bacterial populations decreased more slowly than in traditionally packaged samples, particularly under refrigerated storage. At 4 °C, vacuum-sealed tortillas showed populations between 1 and 2 log CFU/g even after 60 days of storage, whereas traditionally packaged tortillas reached lower population levels in a shorter time. At 25 °C, bacterial populations declined faster than at 4 °C, regardless of the packaging system. Vacuum-sealed tortillas consistently exhibited greater pathogen persistence than traditionally packaged samples. In all treatments, *S.* Typhimurium populations declined throughout storage, exhibiting non-linear inactivation patterns. Accordingly, the Weibull model was selected to describe the survival kinetics because it adequately represented the non-log-linear behavior.

The present study focused on culturable *Salmonella* cells recovered under the enumeration conditions used and did not include specific analyses for sub-lethally injured or viable but non-culturable (VBNC) cells. Therefore, there is the possibility that stressed cells may have remained viable but could not be recovered by the applied culture-based method. We emphasize that this could be a limitation of the present study and propose the assessment of VBNC cells as a complementary method for future research.

### 3.4. Fitting of Survival Kinetics to the Weibull Model

The parameters of the Weibull model estimated to describe the inactivation kinetics of *S.* Typhimurium under different packaging and storage temperature conditions are presented in Table 3. The model showed good agreement with the experimental data, as indicated by the high coefficients of determination (R^2^ = 0.89–0.98) and low RMSE values (0.174–0.501). The estimated initial population (*N*_0_) remained consistent among treatments, ranging from 6.45 to 6.66 log CFU/g, confirming the homogeneity of the inoculum across experimental conditions. Likewise, the first decimal reduction time (δ) varied from 0.67 to 2.57 days but was not significantly affected by either packaging type or storage temperature (*p* > 0.05). Nevertheless, δ tended to increase in vacuum-sealed tortillas stored at 25 °C, indicating a slower initial inactivation under these conditions. In contrast, the Weibull shape parameter (*p*) ranged from 0.37 to 0.53 and was significantly influenced by storage temperature, with higher values at 25 °C than at 4 °C (*p* < 0.05), whereas packaging type had no significant effect. Finally, RMSE values ranged from 0.17 to 0.50, indicating an adequate fit of the Weibull model to the experimental microbial survival data. In all treatments, the *p* parameter remained below 1, indicating upward-concave survival curves characterized by rapid initial reductions followed by increased resistance of the surviving population.

**Figure 2:**
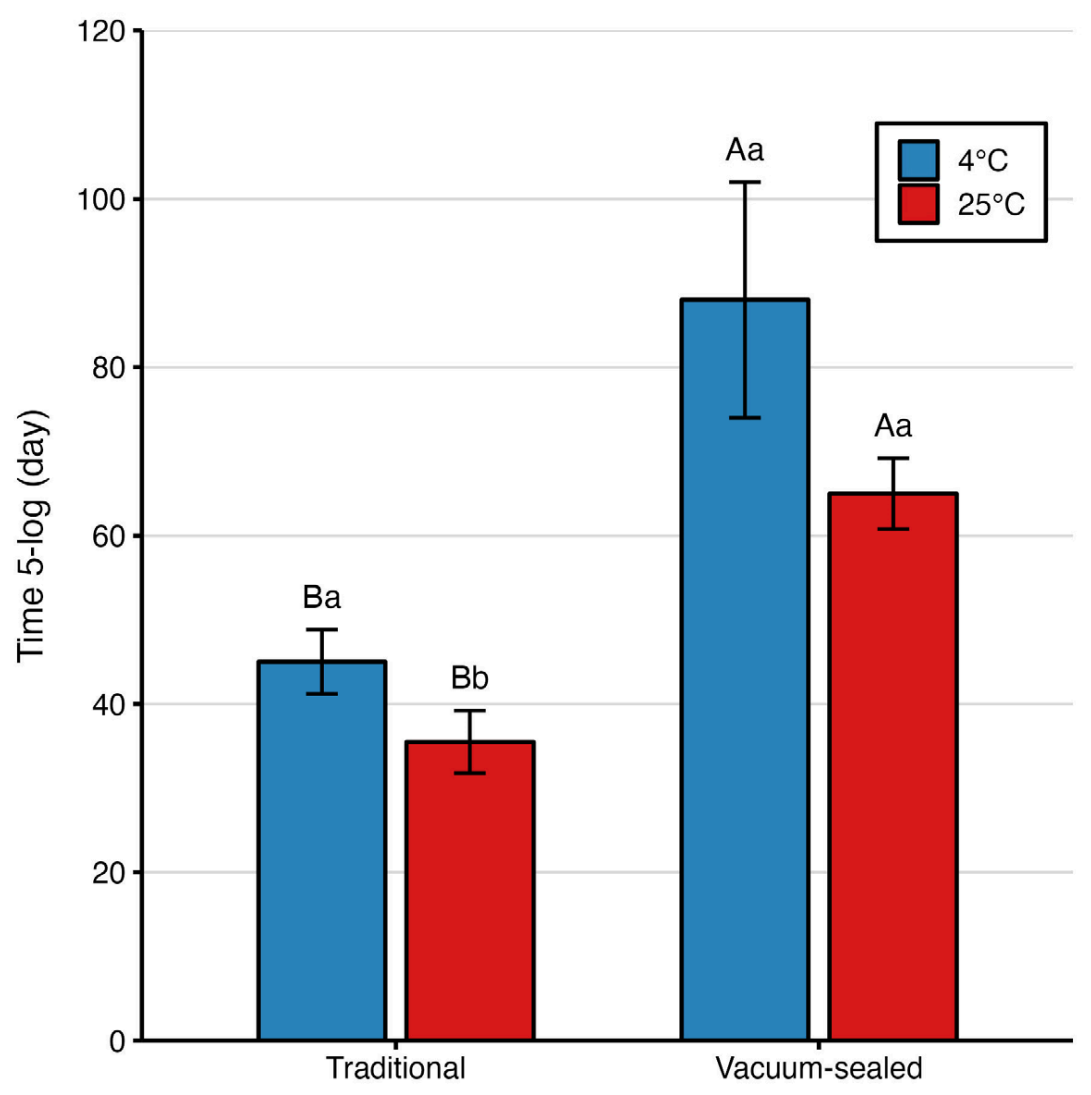
Estimated time required to achieve a 5-log reduction (t_5_) of *S.* Typhimurium in maize tortillas under traditional and vacuum-sealing conditions at different storage temperatures. Bars represent mean values ± standard deviation. Values were calculated from the parameters of the Weibull model fitted to the survival data. Different uppercase letters indicate differences between packaging types at the same storage temperature, whereas different lowercase letters indicate differences between storage temperatures at the same packaging type (*p* ≤ 0.05).

Given the satisfactory fit of the Weibull model, the estimated parameters were subsequently used to calculate the time required to achieve a 5-log reduction (T_5_) of *S.* Typhimurium under each packaging and storage condition. The estimated T_5_ values are presented in Figure 2.

#### Effect Size of Packaging and Temperature Storage on the Weibull Model Parameters That Describe the Inactivation Kinetics of *S.* Typhimurium

The relative contribution of packaging type and storage temperature to the variability of the Weibull model parameters was assessed using effect size statistics (η^2^ and ω^2^) derived from the two-way ANOVA (Table 4). For the δ parameter, packaging type showed a small effect (η^2^ = 0.092; ω^2^ = 0.013), whereas storage temperature exhibited a moderate effect (η^2^ = 0.276; ω^2^ = 0.184). However, neither factor nor their interaction significantly affected δ (*p* > 0.05). The interaction between packaging type and storage temperature explained only a negligible proportion of the total variance (η^2^ = 0.011; ω^2^ = 0.000). For the shape parameter (*p*), storage temperature was the only factor showing a significant effect (F = 7.45, *p* = 0.026), accounting for nearly half of the total variance (η^2^ = 0.471) with a large, adjusted effect size (ω^2^ = 0.383). In contrast, packaging type had a negligible influence on this parameter (η^2^ = 0.016; ω^2^ = 0.000), and no significant interaction between factors was detected (η^2^ = 0.007; ω^2^ = 0.000). The estimated time required to achieve a 5-log reduction (t_5_) was primarily influenced by packaging type, which explained 73.2% of the total variance (η^2^ = 0.732) and showed a very large adjusted effect size (ω^2^ = 0.712). Storage temperature also significantly affected t_5_ (F = 12.75, *p* = 0.007), although its contribution was considerably smaller (η^2^ = 0.150; ω^2^ = 0.137). The interaction between packaging type and storage temperature was not significant and accounted for only a small proportion of the observed variability (η^2^ = 0.024; ω^2^ = 0.012).

## 4. Discussion

The high a_w_ values observed in the industrialized maize tortillas (0.945–0.965) are characteristic of carbohydrate-rich products and may support microbial persistence [17]. The incorporation of hydrocolloids, such as xanthan gum and cellulose gum, may influence water retention and distribution within the matrix. Likewise, the pH values (4.76–5.50), which are lower than those of traditional maize tortillas (~8), are likely associated with the use of additives like organic acids [18]. Although acidification is commonly applied to improve microbial stability, this pH range remains within the tolerance limits of several enteric bacteria, including *Salmonella* and *E. coli*, particularly under reduced oxygen conditions [19]. Overall, variations in moisture content, water activity, and pH may interact to influence microbial growth and survival, thereby affecting the stability and safety of vacuum-sealed maize tortillas [20], suggesting that maize tortillas constitute a heterogeneous matrix capable of supporting microbial persistence through localized microenvironments that modulate stress exposure.

Dietary fiber exhibited the greatest variability among brands, likely reflecting differences in formulation and the incorporation of ingredients such as nopal, inulin, and other vegetable fibers. Soluble fiber can form gel structures by retaining water and entrapping proteins within the food matrix [21,22], thereby influencing moisture retention and product structure. Reported dietary fiber contents in maize tortillas are generally lower than those observed in the present study [23], highlighting the influence of formulation and ingredient selection. However, the addition of fiber-rich ingredients does not necessarily translate into higher dietary fiber contents because processing conditions, ingredient interactions, and fiber solubility also influence the final composition [24]. Discrepancies between declared and measured fiber contents have likewise been reported previously [25]. From a microstructural perspective, this compositional variability emphasizes the spatial heterogeneity of industrialized maize tortillas, generating localized differences in water availability, nutrient distribution, and mechanical constraints that may modulate bacterial physiology and stress adaptation during storage.

The low mold and yeast count observed in most brands may reflect the inhibitory effect of vacuum-sealing on aerobic spoilage microorganisms. Nevertheless, detectable populations in two brands indicate that contamination may have occurred before sealing, possibly due to inadequate hygiene during processing or poor storage conditions of raw materials. Differences in formulation, preservative systems, or packaging integrity may also contribute to the survival of molds and yeasts because these microorganisms can tolerate acidic conditions and reduced oxygen environments. Similar microbial levels have been reported previously for fresh maize tortillas [26]. Likewise, Cabello-Olmo et al. [27] observed that yeast populations in vacuum-sealed cereal-based products declined during the first month of storage and subsequently remained stable for extended periods. These findings suggest that although vacuum-sealing influences microbial dynamics, good manufacturing practices remain the primary factor determining the microbiological stability of packaged tortillas.

Total coliform counts differed among brands and, in several cases, exceeded the Mexican regulatory limit (1.48 log CFU/g), indicating deficiencies in hygienic practices during manufacture [28]. In addition, *Escherichia coli* was detected in three of the nine brands evaluated, indicating non-compliance with Mexican sanitary legislation requiring its absence in ready-to-eat foods. Similar findings have been reported by Castillo et al. [29], who detected total coliforms in 70% and *E. coli* in 32% of freshly prepared tortillas collected in central Mexico. Interestingly, microbial counts in the present study were generally lower than those reported for fresh tortillas, although commercially packaged products would be expected to exhibit even better microbiological quality because of their standardized industrial production. From a matrix perspective, the observed microbial loads indicate that the combination of high aw, moderate acidity, and structural complexity does not prevent microbial survival. Instead, these conditions may allow bacterial populations to persist in a metabolically reduced state. Reduced oxygen availability under vacuum may further decrease oxidative stress, thereby contributing to microbial persistence. Consequently, although vacuum-sealing effectively limits aerobic spoilage organisms, it does not necessarily reduce the survival of facultative or stress-adapted bacteria, emphasizing the importance of matrix–microorganism interactions when evaluating the microbiological stability of cereal-based foods.

The documented occurrence of hygiene indicator microorganisms in commercial tortillas provided the rationale for further evaluating the potential persistence of *S.* Typhimurium under controlled experimental conditions. The frequent co-occurrence of *Salmonella* spp. and *Escherichia coli* in cereal-based and ready-to-eat foods has been previously documented [29]. Accordingly, a controlled challenge study was conducted using laboratory-prepared maize tortillas to evaluate *S.* Typhimurium survival under vacuum-sealed and traditional packaging conditions.

The non-linear survival curves observed throughout storage justified the application of the Weibull model to describe *S.* Typhimurium inactivation. Similar non-log-linear survival patterns and prolonged persistence have been reported in carbohydrate-rich matrices such as wheat flour under different storage conditions [30,31], supporting the use of flexible empirical models to describe pathogen survival in cereal-based products.

The faster reductions observed at 25 °C compared with refrigerated storage suggest that low temperatures favor the persistence of *Salmonella* by reducing metabolic activity and allowing cells to remain viable for extended periods. Moreover, the slower decline observed in vacuum-sealed tortillas indicates that the packaging atmosphere also contributes to pathogen persistence [30]. Reduced oxygen availability may decrease oxidative stress and promote greater cellular stability during storage. Similar effects have been described in vacuum-sealed foods, where reduced oxygen modifies the storage environment and influences *Salmonella* survival under refrigeration [31]. Detectable populations throughout storage demonstrate that *Salmonella* can persist in maize tortillas even under conditions that do not support microbial growth. Under the high-inoculum challenge conditions evaluated in this study, vacuum sealing was associated with slower pathogen inactivation and therefore may prolong *Salmonella* persistence compared with traditional packaging.

Although vacuum-sealing is generally perceived as a strategy to improve microbiological safety, the present results demonstrate that it may instead enhance the long-term survival of foodborne pathogens in cereal-based matrices. Similar behavior has been reported for low-moisture foods such as wheat flour, nuts, and other carbohydrate-rich products, where *Salmonella enterica* remains viable for prolonged periods despite the absence of growth [32]. In these systems, reduced metabolic activity and enhanced stress tolerance contribute to persistence rather than rapid inactivation. However, quantitative information describing pathogen survival in vacuum-sealed maize tortillas remains scarce. Therefore, the present work provides one of the first integrated evaluations combining commercial microbiological profiling, challenge testing, and Weibull modeling to characterize *Salmonella* survival in vacuum-sealed maize tortillas. Collectively, these findings demonstrate that vacuum-sealing, although effective for controlling spoilage microorganisms, may unintentionally prolong pathogen persistence and therefore represent an underrecognized food safety concern in cereal-based products during extended storage.

The analysis of Weibull parameters further supports these observations. The absence of significant differences in the first decimal reduction time (δ) suggests that the initial reduction in *S.* Typhimurium was governed primarily by intrinsic properties of the tortilla matrix rather than by packaging or storage temperature. High water activity, elevated moisture content, pH, nutrient availability, and the structural characteristics of the starch–protein network likely determine the early physiological adaptation of bacterial cells. Similar effects of food matrix properties on pathogen survival have been described previously in cereal-based products [33,34]. Although temperature did not significantly affect δ, its moderate effect size indicates that environmental conditions still contributed to the overall variability in microbial inactivation.

In contrast, storage temperature significantly affected the Weibull shape parameter (*p*), indicating that temperature modified the curvature of the survival curves and therefore the distribution of resistant bacterial subpopulations. Previous studies have similarly shown that refrigeration prolongs *Salmonella* survival in wheat flour and cornmeal compared with ambient storage [14], supporting the interpretation that lower temperatures modify microbial resistance patterns during storage.

Packaging type exerted the greatest influence on the estimated time required to achieve a 5-log reduction (t_5_), indicating that the storage atmosphere was the primary determinant of long-term pathogen persistence. Reduced oxygen availability under vacuum conditions likely decreases oxidative stress and enhances cellular stability, thereby extending survival. Similar observations have been reported by Djordjević et al. [35], who found higher *Salmonella* populations in vacuum-packaged minced meat than under modified-atmosphere packaging with elevated CO_2_ concentrations. These findings reinforce the importance of considering packaging atmosphere, together with storage temperature, when evaluating the microbiological safety and shelf life of vacuum-sealed maize tortillas.

The very large effect size observed for packaging type on t_5_ (η^2^ = 0.732; ω^2^ = 0.712) indicates that packaging atmosphere was the predominant factor governing long-term pathogen persistence. Unlike statistical significance alone, effect size quantifies the practical relevance of experimental factors, demonstrating that oxygen availability had a substantially greater influence on *Salmonella* survival than storage temperature. This finding reinforces the importance of packaging atmosphere as a critical variable in predictive microbiology and microbial risk assessment of vacuum-packaged cereal-based foods.

## 5. Conclusions

This study demonstrates that vacuum-sealing does not ensure the inactivation of *S.* Typhimurium in maize tortillas and may prolong pathogen survival, particularly under refrigeration, challenging the assumption that vacuum packaging enhances microbiological safety. Microbial persistence was governed by the interaction between packaging conditions, storage temperature, and intrinsic matrix characteristics such as a_w_, pH, and structural heterogeneity. From a food safety perspective, these findings indicate that vacuum-sealing alone should not be considered a stand-alone intervention for controlling *S.* Typhimurium when post-process contamination occurs. Furthermore, maize tortillas may serve as a useful cereal-based food model for evaluating pathogen survival dynamics under different storage and packaging conditions.

## Figures and Tables

**Table 1:** Proximate composition, a_w_, and pH of vacuum-sealed maize tortillas.

Brand	Moisture Content (%)	a_w_	pH	Protein	Fat	Dietary Fiber	Ash	Carbohydrates
				g/100 g				
V1	43.21 ± 1.05 ^c^	0.958 ± 0.002 ^abc^	4.85 ± 0.01 ^de^	8.81 ± 0.41 ^ab^	1.99 ± 0.08 ^c^	12.68 ± 0.12 ^b^	1.65 ± 0.10 ^c^	87.55 ± 0.31 ^ab^
V2	44.77 ± 1.22 ^bc^	0.962 ± 0.004 ^ab^	4.76 ± 0.04 ^e^	7.99 ± 0.51 ^bc^	3.20 ± 0.07 ^a^	6.77 ± 0.48 ^e^	2.72 ± 0.03 ^a^	86.10 ± 0.46 ^c^
V3	35.56 ± 0.70 ^e^	0.960 ± 0.003 ^ab^	5.11 ± 0.01 ^c^	8.50 ± 0.23 ^abc^	2.56 ± 0.10 ^b^	20.17 ± 0.58 ^a^	2.17 ± 0.02 ^b^	86.76 ± 0.18 ^bc^
V4	39.65 ± 0.57 ^d^	0.965 ± 0.008 ^a^	5.31 ± 0.01 ^b^	8.95 ± 0.56 ^ab^	1.01 ± 0.10 ^d^	3.74 ± 0.34 ^f^	2.17 ± 0.09 ^b^	87.87 ± 0.73 ^a^
V5	37.09 ± 0.43 ^e^	0.945 ± 0.005 ^c^	5.03 ± 0.01 ^c^	9.32 ± 0.20 ^a^	1.76 ± 0.04 ^c^	9.52 ± 0.39 ^d^	2.70 ± 0.02 ^a^	86.21 ± 0.18 ^c^
V6	46.02 ± 0.84 ^b^	0.961 ± 0.003 ^ab^	4.82 ± 0.01 ^de^	8.16 ± 0.17 ^bc^	0.98 ± 0.07 ^d^	9.76 ± 0.14 ^cd^	2.70 ± 0.05 ^a^	88.15 ± 0.16 ^a^
V7	46.92 ± 0.84 ^ab^	0.957 ± 0.002 ^abc^	5.09 ± 0.02 ^c^	8.38 ± 0.19 ^abc^	1.04 ± 0.01 ^d^	7.59 ± 0.62 ^e^	2.66 ± 0.09 ^a^	87.91 ± 0.14 ^a^
V8	44.80 ± 0.26 ^bc^	0.949 ± 0.010 ^bc^	5.51 ± 0.08 ^a^	7.70 ± 0.33 ^c^	2.54 ± 0.14 ^b^	11.49 ± 0.58 ^b^	3.03 ± 0.21 ^a^	86.74 ± 0.40 ^bc^
V9	49.09 ± 1.28 ^a^	0.957 ± 0.004 ^abc^	4.89 ± 0.06 ^d^	9.19 ± 0.26 ^a^	1.16 ± 0.15 ^d^	11.22 ± 0.94 ^bc^	2.91 ± 0.26 ^a^	86.75 ± 0.32 ^bc^

Results are expressed as the mean ± standard deviation of three replicates and were calculated on a dry-weight basis. Different letters indicate significant differences among brands according to the Tukey test (α = 0.05).

**Table 2:** Content of indicator microorganisms in vacuum-sealed tortillas.

Brand	Log CFU/g		
	Aerobic Plate Count (APC)	Total Coliforms (TC)	Molds and Yeasts (M&Y)
V1	2.04 (1.48–2.89) ^ab^	1.79 (1.60–2.00) ^ab^	<1.00 (<1.00–1.60) ^ab^
V2	2.30 (1.15–3.19) ^ab^	1.44 (1.00–2.10) ^ab^	<1.00 (<1.00–2.31) ^ab^
V3	2.38 (1.95–3.80) ^ab^	2.02 (1.00–2.50) ^ab^	<1.00 (<1.00–1.60) ^ab^
V4	2.88 (2.11–4.46) ^ab^	2.24 (1.30–5.26) ^a^	<1.00 (<1.00–1.53) ^ab^
V5	2.03 (1.30–2.90) ^ab^	2.15 (1.40–4.48) ^a^	<1.00 (<1.00–2.33) ^ab^
V6	2.08 (1.48–2.43) ^b^	2.44 (2.10–3.50) ^a^	<1.00 (<1.00–1.60) ^ab^
V7	2.43 (1.34–3.10) ^ab^	1.60 (1.10–2.98) ^ab^	<1.00 (<1.00–2.40) ^ab^
V8	2.87 (2.65–3.01) ^ab^	1.20 (1.00–1.60) ^bcd^	1.15 (1.00–1.87) ^a^
V9	2.51 (1.78–2.89) ^ab^	2.13 (1.50–2.60) ^ab^	1.30 (1.00–1.98) ^a^

Each value represents the median and range of six independent samples. For each group of microorganisms, different letters indicate a significant difference between brands according to Dunn’s test (α = 0.05). APC: aerobic plate count; TC: total coliforms; M&Y: molds and yeasts.

**Table 3:** Weibull model parameters describing the inactivation kinetics of *S.* Typhimurium in maize tortillas under different packaging and storage conditions.

Packaging Type	Storage Temperature (°C)	*N*_0_ ± SD (log CFU/g)	δ ± SD (d)	*p* ± SD	RMSE	R^2^
Traditional	4	6.58 ± 0.16 ^Aa^	0.67 ± 0.29 ^Aa^	0.38 ± 0.05 ^Ab^	0.17	0.98
	25	6.45 ± 0.08 ^Aa^	1.64 ± 0.29 ^Aa^	0.53 ± 0.05 ^Aa^	0.31	0.96
Vacuum-sealed	4	6.64 ± 0.10 ^Aa^	1.13 ± 0.17 ^Aa^	0.37 ± 0.02 ^Ab^	0.50	0.89
	25	6.66 ± 0.19 ^Aa^	2.57 ± 2.15 ^Aa^	0.49 ± 0.15 ^Aa^	0.32	0.95

*N*_0_: initial population (log CFU/g); δ: time required for the first decimal reduction (days); *p*: shape parameter of the survival curve; RMSE: root-mean-square error; R^2^: coefficient of determination. Values represent mean ± standard deviation. Different uppercase letters indicate differences between packaging types at the same storage temperature, and lowercase letters indicate differences between storage temperatures at the same packaging type according to Tukey’s test (*p* < 0.05).

**Table 4:** Two-way ANOVA evaluating the effects of packaging type and storage temperature on Weibull model parameters describing the inactivation kinetics of *S.* Typhimurium.

Variable	Source	df	SS	MS	F	*p*-Value	η^2^	ω^2^
*δ*	Packaging type	1	1.449	1.449	1.18	0.309	0.092	0.013
	Storage temperature	1	4.351	4.351	3.55	0.096	0.276	0.184
	Interaction	1	0.167	0.167	0.14	0.722	0.011	0.000
	Residuals	8	9.815	1.227	—	—	—	—
*p*	Packaging type	1	0.00175	0.00175	0.26	0.626	0.016	0.000
	Storage temperature	1	0.05083	0.05083	7.45	0.026	0.471	0.383
	Interaction	1	0.00078	0.00078	0.11	0.743	0.007	0.000
	Residuals	8	0.05460	0.00682	—	—	—	—
t_5_	Packaging type	1	3884.401	3884.401	62.26	<0.001	0.732	0.712
	Storage temperature	1	795.441	795.441	12.75	0.007	0.150	0.137
	Interaction	1	128.708	128.708	2.06	0.189	0.024	0.012
	Residuals	8	499.120	62.390	—	—	—	—

The main effects and/or interaction of main effects were significant (*p*-value ≤ 0.05). SS: sum of squares; MS: mean square; η^2^: eta-squared; ω^2^: omega-squared effect size.

## Data Availability

The original contributions presented in the study are included in the article; further inquiries can be directed to the corresponding author.
